# Does glaze firing affect the strength of advanced lithium disilicate after simulated defects?

**DOI:** 10.1007/s00784-023-05246-1

**Published:** 2023-09-20

**Authors:** Yuqing Lu, Amanda Maria de Oliveira Dal Piva, João Paulo Mendes Tribst, Albert J Feilzer, Cornelis J Kleverlaan

**Affiliations:** 1 Department of Dental Materials Science, Academic Centre for Dentistry Amsterdam (ACTA), Universiteit Van Amsterdam and Vrije Universiteit, Gustav Mahlerlaan 3004, 1081 LA Amsterdam, Noord-Holland The Netherlands; 2 Department of Reconstructive Oral Care, Academic Centre for Dentistry Amsterdam (ACTA), Universiteit Van Amsterdam and Vrije Universiteit, Amsterdam, The Netherlands

**Keywords:** Lithium disilicate, Glass–ceramics, Crystallization, Flexural strength, Dental clinics

## Abstract

**Objective:**

To study the influence of glazing on strength repair of lithium disilicate glass–ceramics after defect incorporation in different production processing phases.

**Materials and methods:**

Bar-shaped specimens (1 × 1 × 12 mm, *n* = 280; 20/group) made from different lithium disilicate ceramics (IPS e.max CAD, Ivoclar, “LD” or advanced lithium disilicate CEREC Tessera, Dentsply Sirona, “ALD”) were exposed to 7 different protocols: crystallized without (*c*) and with glaze layer (*cg*), with a defect incorporated before crystallization without (*ic*) and with glaze layer (*icg*), with a defect after crystallization without (*ci*) or with glaze layer (*cig*), and defect incorporated after the glaze layer (*cgi*). The flexural strength was determined using the three-point bending test. Analysis of indented areas and fractured specimens was performed by scanning electron microscopy. Flexural strength data were evaluated by two-way ANOVA followed by Tukey tests (*α* = 5%).

**Results:**

Two-way ANOVA revealed a significant influence of ceramic (*p* < 0.001; *F* = 55.45), protocol (*p* < 0.001; *F* = 56.94), and the interaction protocol*ceramic (*p* < 0.001; *F* = 13.86). Regardless of ceramics, defect incorporation as final step resulted in the worst strength, while defects introduced before crystallization did not reduce strength. Glaze firing after defect incorporation led to strength repair for ALD, whereas such an effect was not evident for LD.

**Conclusions:**

The advanced lithium disilicate must receive a glaze layer to achieve its highest strength. Defects incorporated in the pre-crystallized stage can be healed during crystallization. Defects should not be incorporated after glazing.

**Clinical relevance:**

Clinical adjustments should be performed on pre-crystallized or crystalized restorations that receive a glazer layer afterwards.

## Introduction

Reinforced glass–ceramics have passed through improvements leading to materials that combine sufficient strength and esthetics. These biomimetic characteristics have been missed for a long time in the first generation of zirconia and glass–ceramics, respectively. However, with the contemporary processing methods and material composition, it is possible to successfully rehabilitate damaged teeth using these synthetic materials. In addition, glass–ceramics present a high amount of glassy phase that allows its surface conditioning with acid etching promoting suitable bond strength to different substrates [1].

One of the most common reinforced glass–ceramic materials available for dental application is lithium disilicate reinforced glass–ceramic (LD). LD can be milled or heat-pressed in different restoration designs, making it widely popular for many dental applications, e.g., anterior and posterior crowns, implant-supported prosthesis, abutments, inlays, onlays, occlusal veneers, veneers, and ultrathin veneers [2]. LD restoration showed a high success rate of more than 90% in short- and mid-term observation [3–5]. However, long-term studies of 15 years reveal lower survival rates regarding single crown (81.9%) [6], posterior three-unit fixed prostheses (48.6%) [7], and posterior inlay-retained prostheses (22%) [8]. Specifically, fracture emerged as the main cause of failure at the later stage, according to studies on lithium disilicate restorations manufactured from IPS e.max Press developed by Ivoclar [7, 8].

Every clinician aims for a restorative material that combines suitable mechanical and optical properties to mimic the natural teeth. This is possible for LD that is available in different translucencies and has the indication to rehabilitate innumerous clinical situations. However, during the oral try-in of the restoration, adjustments are sometimes necessary [9]. For LD, this procedure can be done before [10] and after [3] final crystallization, including internal and marginal adaptation, proximal contact, and occlusion. Usually, diamond burs are indicated to adjust the restoration, while silicone diamond burs can be used as the following step to polish the adjusted area, since it is well-known that the surface roughness is directly related to biofilm formation [11] and slow crack growth when the ceramic is submitted to fatigue in the oral medium [12]. Other clinicians opt to prove the restoration and perform grinding adjustments and the cementation procedure in a single-time visit [13]. However, it is not well-known if there is any deleterious effect in performing the adjustments after crystallization or glazing, which raises the question of which moment the restoration adjustment procedure should be performed when necessary not to decrease the material’s strength. In addition to the clinical adjustment by dentists, there are other possible situations in which defects can be incorporated in the ceramic surface, e.g., the milling process (before crystallization) [14] and support removal after milling (before crystallization) [15], as well as chewing and biting during a long period of service (after the glaze firing) [16]. Therefore, it is worthwhile to understand the effect of defect incorporation moment on the mechanical behavior of restorations.

To overcome the deleterious effect of surface defects, an advanced lithium disilicate (ALD) has been developed with the claim that possible surface defects or hairline fractures could be healed after a short crystallization firing [17]. Compared with conventional LD, ALD shows a different phase composition after mandatory firing, including 40% less Li_2_Si_2_O_5_, approximately twice as much residual glass and the presence of quartz-like phases (3.7 vol%) [18]. In addition, a smaller microstructural size was observed for ALD after acid etching [19], indicating a potentially significant difference in their mechanical performance. However, the relevant literature on the properties of this new material in comparison with conventional LD is currently limited. Moreover, according to the manufacturer, a glaze layer is necessary for the firing process to achieve its final strength. As a consequence, clinical adjustments are recommended to be performed after glazing.

Flexural strength is an essential mechanical property for dental ceramic materials. It determines whether the restoration can withstand occlusal forces and plays a critical role in the survival of restorations. Based on the strength test, indentation strength is another important technique to understand the mechanical properties of ceramic materials [10]. It incorporates controlled flaws with standardized shape and size and eliminates the influence of other defects on the results [20]. In this study, a Vickers indenter was used to introduce the flaw and simulate the surface damage in the restoration at various moments simulating different situations: before crystallization, after crystallization and before the glaze firing, and after the glaze firing. Therefore, this study was aimed at assessing the influence of defect incorporation and glazing on the flexural strength of two lithium disilicates. The null hypothesis is that the moment of defect incorporation would not affect the flexural strength of LD and ALD regardless of glaze firing.

## Materials and methods

### Specimen’s preparation

**Fig. 1:**
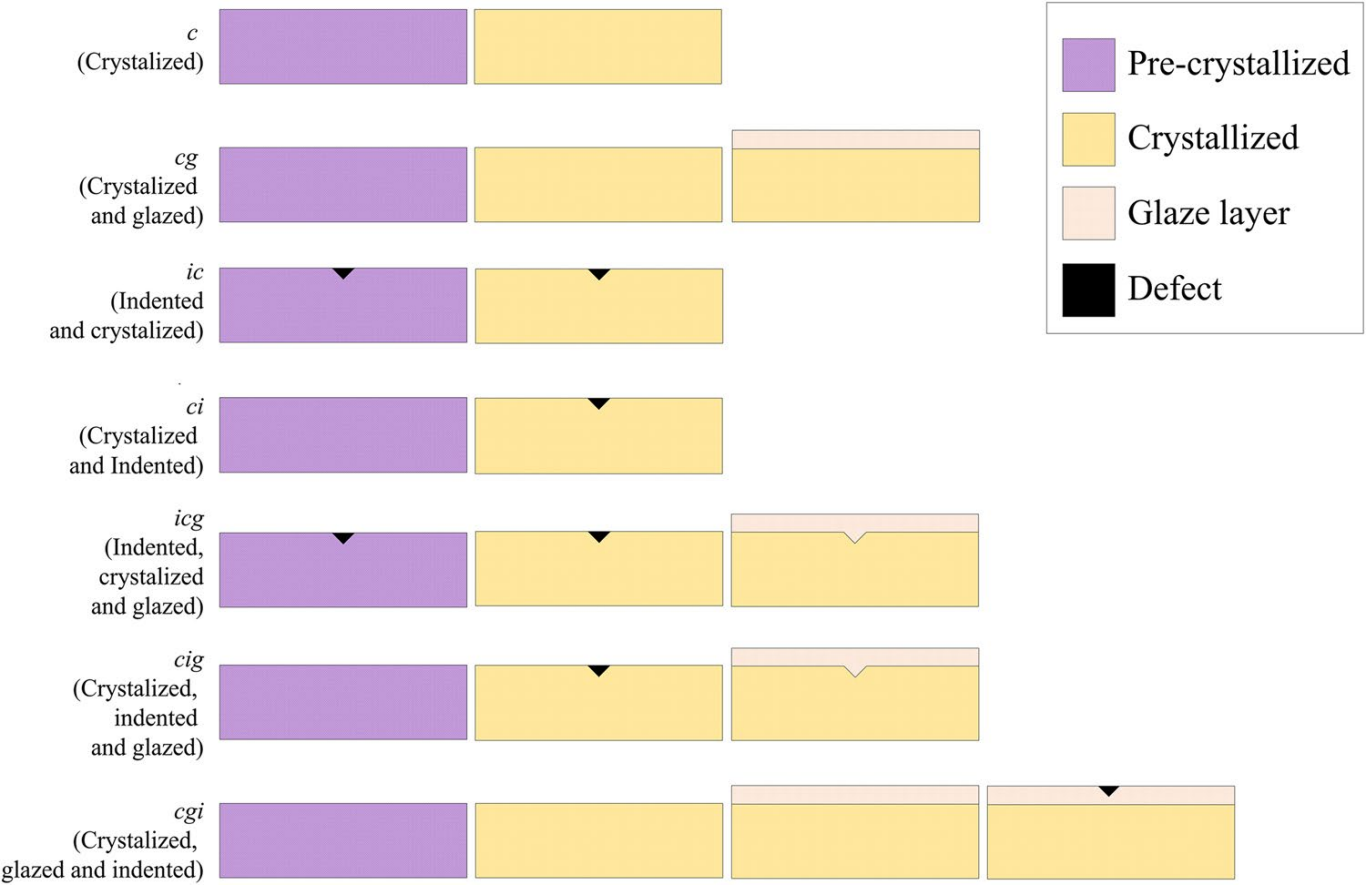
The study’s design of five protocols to simulate different moments for defect incorporation (before or after crystallization and/or glaze firing) compared to two situations without defect incorporation. The 1st and 2nd protocols were respectively conventional crystallization firing without (*c*) and with a glaze layer (*cg*) without defect incorporation. In the 3rd and 4th protocols, the defect was incorporated before crystallization firing, while one group did not receive a glaze layer (*ic*) and the other received (*icg*). The 5th and 6th groups received a defect after crystallization firing, without (*ci*) or with a glaze layer (*cig*) in sequence. Finally, the latest condition is simulated defects incorporated after the glaze layer application (*cgi*). All protocols have been simulated for both materials (LD and ALD)

One hundred and forty bar-shaped specimens (1 × 1 × 12 mm ± 0.2) [21–23] were obtained from each lithium disilicate glass–ceramic: “IPS e.max CAD” (LD; batch number: Y52153; Ivoclar, Lichtenstein) and “CEREC Tessera” (ALD; batch number: 16011535; Dentsply Sirona, Germany) using a diamond-coated saw in a precision cutting machine (IsoMet 1000; Buehler, Lake Bluff, IL). All specimens were polished until the final dimension using SiC abrasive papers (Ecomet; Buehler Ltd., Evanston, IL) with grit sizes of P800 and P1200. Then, the specimens were submitted to different clinical simulations summarized in Fig. 1. The brand names of all the materials and their compositions are presented in Table 1, and the firing protocols including mandatory firing of the evaluated CAD/CAM lithium disilicate glass–ceramics as well as the glazing procedure are shown in Table 2.

**Table 1:** Materials, trade names, manufacturers and composition

Material and trade name	Manufacturers	Composition (wt%)
Lithium disilicate glass–ceramic (LD), IPS e.max CAD	Ivoclar, Schaan, Liechtenstein	SiO_2_, 57.0—80.0% Li_2_O, 11.0—19.0% K_2_O, 0.0—13.0% P_2_O_5_, 0.0—11.0% Other oxides
Advanced lithium disilicate (ALD), CEREC Tessera	Dentsply Sirona, Hanau-Wolfgang, Germany	Glass zirconia matrix Lithium disilicate Virgilite (LiAlSiO_6_)
Glaze material, Universal Spray Glaze	Dentsply Sirona, Hanau-Wolfgang, Germany	Silicate glass, isopropyl alcohol, and isobutane propellant

**Table 2:** Firing protocols for lithium disilicate (LD), advanced lithium disilicate (ALD), and glazing

Material	*B* (min)	*S* (*°C*)	*t*_1_ (°C/min)	*T*_1_ (°C)	*H*_1_ (s)	*t*_2_ (°C/min)	*T*_2_ (°C)	*H*_2_ (s)	*L* (°C)	Vacuum 1 (°C)		Vacuum 2 (°C)	
										*V*_1_	*V*_2_	*V*_1_	*V*_2_
LD	6	403	60	770	10	30	850	600	700	550	770	770	850
ALD	2	403	55	760	120	/	/	/	/	/	/	/	/
Glazing	2	400	55	760	120	/	/	/	/	/	/	/	/

*B* (min) = closing time, *S* (°C) = standby temperature, *t* (°C/min) = heating rate, *T* (°C) = firing temperature, *H* (s) = holding time, *L* (°C) = long-term cooling, *V*_1_ (°C) = vacuum-on temperature, *V*_2_ (°C) = vacuum-off temperature

To simulate the incorporation of a defect, an indentation was performed in the middle of the tensile side of each specimen, using a Vickers indenter with a 19.6 N load for 15 s (HM-124 Hardness Testing Machine, Mitutoyo Corp., Kanagawa, Japan). Considering the length of the generated damage, the defect length and depth had an average size of 73 µm and 14 µm, respectively.

### Flexural strength

Twenty specimens (*n* = 20) from each group were submitted to a 3-point bending test with a universal testing machine (Instron 6022; Instron Limited, High Wycombe, UK). The load was applied on the middle of the surface using a load cell (1000 N) with a crosshead speed of 0.5 mm/min. Flexural strength (MPa) was calculated according to the following equation based on ISO 6872–2015:

$${\sigma }_{u}=\frac{3Pl}{2b{h}^{2}}$$

where *P* is the maximum load (in N), *l* is the distance between the two supports (in mm), *b* represents the specimen width (in mm), and *h* is the specimen thickness (in mm).

### Scanning electron microscopy (SEM)

All the fractured specimens were submitted to the fractographic analysis. The fractured surfaces were examined under SEM (EVO LS15; Carl Zeiss, Oberkochen, Germany) at × 165 and × 500 magnifications to identify the fracture origin. In addition, representative specimen per group was also evaluated under SEM at × 1000 magnification for the indented area. All the specimens received a gold coat in a low-pressure atmosphere using an ion sputter coater.

### Data analysis

Flexural strength data were submitted to one- and two-way analyses of variance (ANOVA) followed by Tukey tests (*α* = 5%). One-way ANOVA was applied to compare the mean values of all subgroups within each material. Two-way ANOVA was used to evaluate the effect of materials and protocols on strength as well as the interaction between factors. In addition, the strength data were also used to identify the Weibull distribution for each group using maximum likelihood method with 95% confidence interval.

## Results

### Flexural strength

**Fig. 2:**
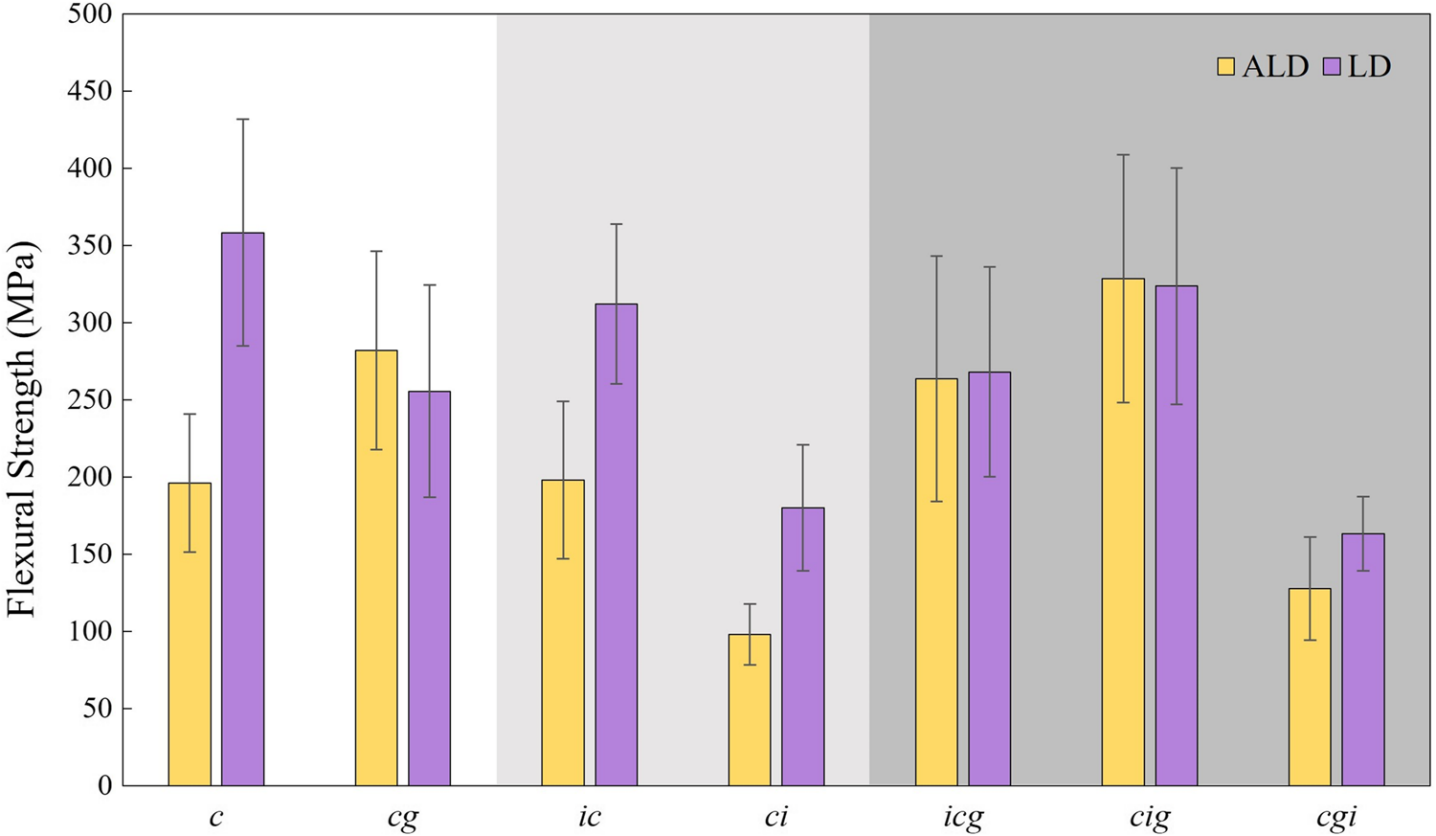
Mean flexural strength and standard deviation according to the ceramic material and moment of defect incorporation

Table 3 and Fig. 2 present the mean flexural strength value and standard deviation of each group according to each material. Two-way ANOVA revealed a significant influence caused by ceramic (*p* < 0.001; *F* = 55.45) and protocol (*p* < 0.001; *F* = 56.94) ceramics. In addition, the grouping comparing all groups is presented since the interaction protocol*ceramic has also presented statistical significance (*p* < 0.001; *F* = 13.86).

**Table 3:** Groups’ distribution according to ceramic material and crystallization firing with and without simulated defects at different moments and flexural strength (MPa) results for each material and for the interaction protocol*ceramic

Groups	Protocol	Flexural strength	Grouping
LD*c*	Crystallized	358.2 ± 73.5	A
LD*cg*	Crystallized + glaze firing	255.4 ± 68.7	DE
LD*ic*	Indented + crystallized	312 ± 51.9	ABCD
LD*ci*	Crystallized and indented	180 ± 40.9	FG
LD*icg*	Indented + crystallized + glaze firing	268 ± 68	BCD
LD*cig*	Crystallized + indented + glaze firing	323.6 ± 76.5	ABC
LD*cgi*	Crystallized + glaze firing + indented	163.2 ± 24.1	FG
ALD*c*	Crystallized	195.9 ± 44.8	EF
ALD*cg*	Crystallized + glaze firing	282 ± 64.3	BCD
ALD*ic*	Indented and crystallized	198 ± 50.9	EF
ALD*ci*	Crystallized and indented	97.8 ± 19.7	H
ALD*icg*	Indented and crystallized + glaze firing	263.6 ± 79.6	CD
ALD*cig*	Crystallized indented and glaze firing	328.4 ± 80.1	AB
ALD*cgi*	Crystallized + glaze firing and indented	127.6 ± 33.3	GH

Different small letters correspond to statistical differences between procedures for the same ceramic. And different capital letters mean difference between all evaluated groups

Based on Tukey results, considering the strength of unaffected materials during production phases, crystallized LD is stronger than ALD (*p* < 0.001). After glazing, LD became weaker (*p* < 0.001) while ALD strength increases (*p* < 0.001). There was no significant difference between ALD*cg* and LD*cg* (*p* = 0.154). Regarding the influence of the defect incorporation moment in the production process, the defect incorporation as the final step (*ci*, *cgi*) resulted in the worst strength for both ceramics. However, no difference was found between ALD*cgi* and LD*cgi* (*p* = 0.057). In addition, defects introduced before crystallization (*ic*) did not reduce the crystallized materials’ strength for LD (*p* = 0.290) and ALD (*p* = 1.000). Observing the glaze effect, ALD*cig* was stronger than the corresponding groups without glaze firing (ALD*ci*, *p* < 0.001) and similar to ALD*cg* (*p* = 0.282); ALD*icg* was also stronger than ALD*ic* (*p* = 0.011) and similar to ALD*cg* (*p* = 1.000). For LD*cg*, the similarity was found with *ic* and *icg* (*p* = 0.396).

**Fig. 3:**
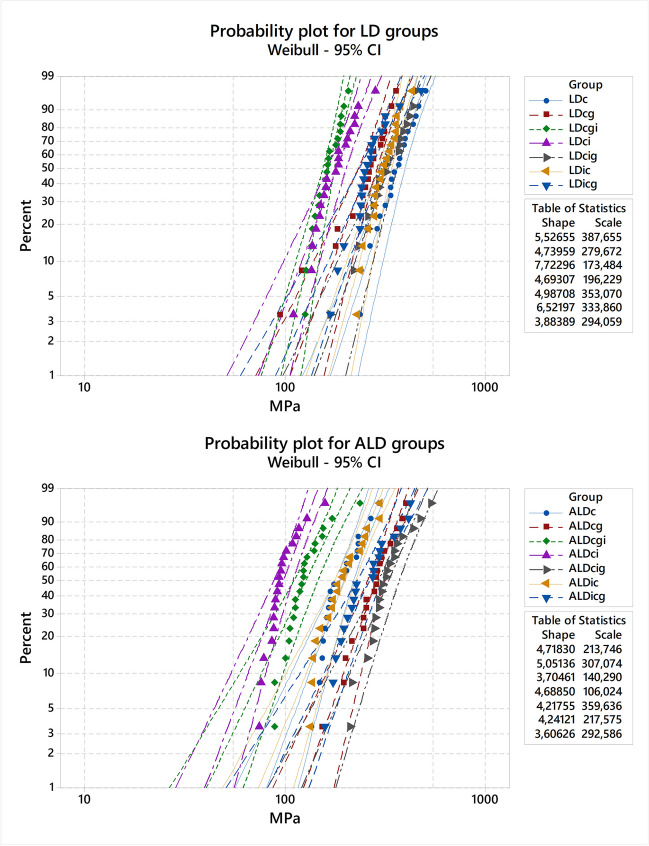
Weibull 95% confidence interval (CI) plots for LD (**A**) and ALD (**B**) groups

According to Weibull 95% CI (Fig. 3), it was possible to observe that LD groups were more sensitive to the simulated protocols when compared to ALD groups since the Weibull modulus of LD ranged from 3.6 to 5.5 while that of ALD ranges from 3.8 to 7.7. The probability plot revealed that ALD*cg* and LD*ic* presented the highest reliability for flexural strength values, while *icg* protocol showed the lowest reliability for both materials.

### Fractography

**Fig. 4:**
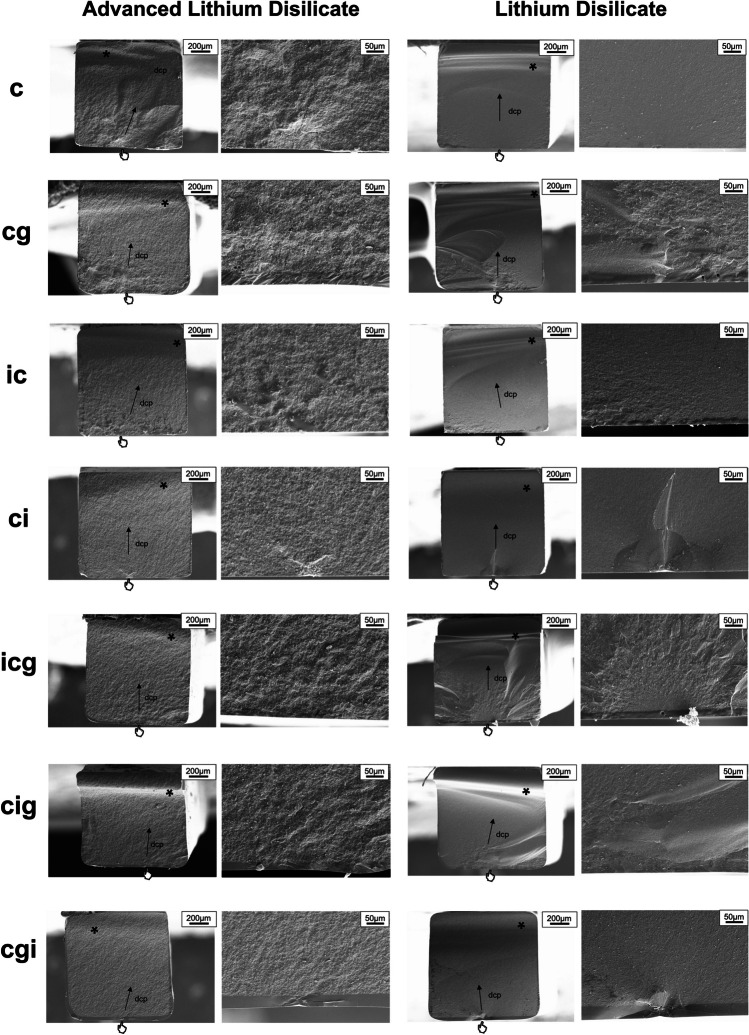
Fractographic analysis of representative failed specimens. The images on the left (× 165) show the complete view of the failed specimen, indicating the failure origin on the tensile side (positioned as the top side in the figures). The figures on the right (× 600) show an approximated view of the failure origin. The black arrows indicate the direction of crack propagation (dcp), the asterisks indicate the compression curl, and the hands indicate the fracture origins

According to the fractographic analysis, the defects were not visible when the indentation was performed before crystallization (*ic*) or crystallization and glaze layer application (*icg* and *cig*), while in groups in which the defect was incorporated as the last step, the defect was clearly visible (*ci* and *cgi*). Finally, the analysis revealed that all specimens failed from the tensile side (Fig. 4).

### Indented areas

**Fig. 5:**
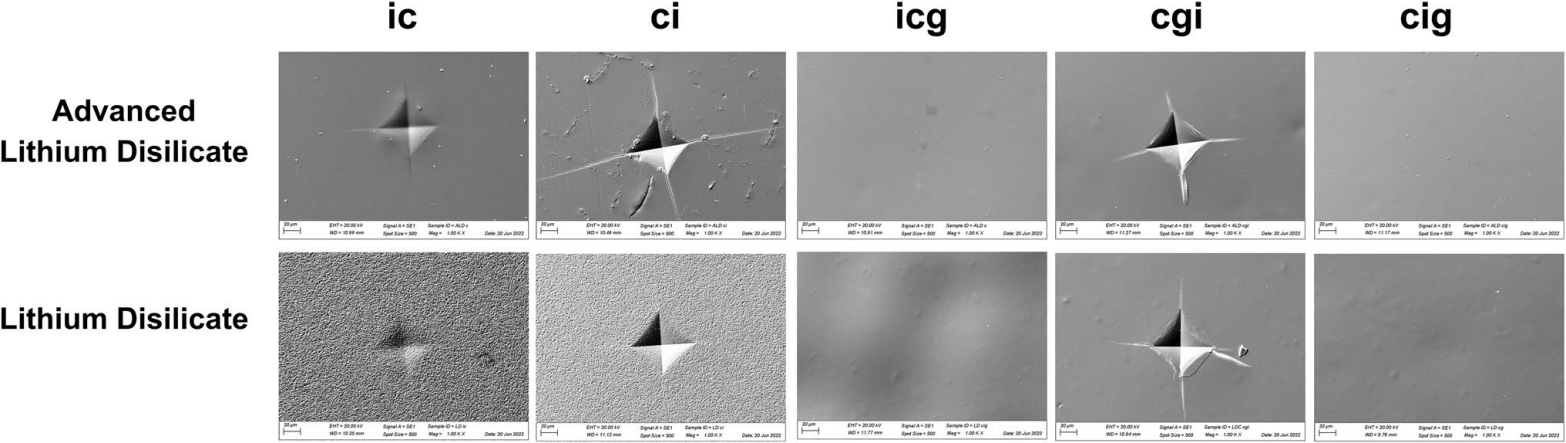
SEM image (× 1000) of the indented areas according to the moment of defect incorporation

Figure 5 exhibits the five protocols for defect incorporation. According to the non-indented areas of *ic* and *ci*, ALD showed a smoother surface than LD after crystallization. Protocols *cgi*, *icg*, and *cig* showed the glossy surface effect of glazing. The defect incorporation, directly after crystallization or after applying a glaze layer, promoted flaw formation. For protocol *ci*, the indentations showed similar diagonal lengths but different radial crack lengths. For group *cgi*, since the same glaze layer was applied for both materials, the indentations of ALD and LD presented similar diagonal and radial crack lengths. These images of *ci* and *cgi *also revealed a comparison between the mechanical properties of ALD and LD after glaze firing. For both materials, the indentation from group *ic* had vague boundaries with fewer/shorter cracks, revealing that the damage caused by the indentation is minimized/healed after the crystallization firing. However, the indentations of *icg* and *cig* were covered by the glaze layer.

## Discussion

The aim of this study was to characterize the flexural strength of two lithium disilicates and assess the effect of defect incorporation moments and glaze firing. Based on the results, LD and ALD showed different flexural strengths, which are affected by the moment of defect incorporation as well as glaze firing. Therefore, the null hypothesis was rejected. In this study, a Vickers indentation with a load of 19.6 N was used to simulate damage by clinical adjustments, which consists of brittle fracture and plastic deformation [14]. Only a small defect caused an approximately 50% decrease of the average flexural strength for both LD and ALD, which indicates the sensitivity of ceramics to such type of defects, as well as the effectiveness of the defect incorporation method for evaluating the influence of incorporation moment and glazing firing.

The lithium disilicates evaluated in this study presented different flexural strength under as-crystallized condition (ALD*c* and LD*c*). The mean initial strength of ALD was calculated as 195.9 MPa, which is close to another study also adopting the 3-point bending [15] but much lower than a biaxial test [16]. Since the effective volume/area of uniaxial tests like 3-point bending is smaller than that of biaxial tests, the former usually obtains higher flexural strength values than the latter [24]. The explanation could be that the uniaxial tests are more susceptible to edge defects incorporated during specimen preparation, while such edge damage is also common in restorations by milling. Additionally, it can be seen in Fig. 5 that the Vickers indenter generated similar indentations on both materials but shorter crack lengths for LD*c*, revealing that both materials might have similar hardness but different capabilities against crack propagation. This is consistent with another study [25] that LD has a higher fracture toughness of 2.04 MPam^1/2^ in comparison with ALD (1.45 MPam^1/2^), which also contributes to the higher flexural strength of LD.

According to the results, defect incorporation moment had a significant influence on flexural strength. For both materials, group *cgi* and *ci* produced the worst strength. This is consistent with the finding in Fig. 4 that the fracture origins of *ci* and *cgi* were the introduced defects located in the middle of the tensile surface, while for other groups, the failure started from the flaws in the tensile surface within both materials and glaze layer. For both ceramics, group *ic* generated similar strength as group *c*, indicating that the defect incorporation before crystallization did not influence the strength. This is in agreement with fractography that the incorporated defects failed to dominate the fracture initiation. It is possible that the crystallization process can heal the flaws so that the incorporated defect size was reduced. Evidence can be found in Fig. 4, that the defects showed vague boundaries, and the radial cracks were reduced or shortened. Such a phenomenon where the induced pre-crack on pre-crystallized LD failed to control the fracture after fully crystallization has also been previously reported [26].

The effect of glaze on strength recovery varies between the evaluated materials. For ALD, groups *cg*, *icg*, and *cig* were significantly stronger than groups *c*, *ic*, and *ci* respectively, and both *icg* and *cig* even generated similar strength as *cg*. This indicates that glaze firing after defect incorporation either before or after crystallization led to full strength recovery for ALD, while it did not have the same repair effect on LD. As a result, the flexural strength of both lithium disilicates after the glaze firing (LD*cg*, ALD*cg*) was similar. A possible explanation is that the incorporated defects and other surface flaws of ALD might be healed after the glaze firing, whereas this did not happen to LD. Glass–ceramics can benefit from auto-glaze to obtain a smooth surface and improve its mechanical properties when getting refired, during which the glass component filled the surface flaws and reduced their depth and sharpness [27]. A recent study [22] found that even an additional firing cycle without glaze material (same protocol as normal glaze firing) can also generate a higher flexural strength of ALD than a single mandatory firing, although this improvement is not as great as mandatory firing followed by glazing, indicating that a healing effect of glaze firing on ALD is attributed to both the glaze material and the refiring process. However, such a glaze firing cycle is not sufficient to heal the defects in LD [28, 29], and the application of glaze material even generates additional defects within the glaze layer that have a larger size than the defects within LD [22].

This study showed that both materials had similar strength after glazing and surface damage by clinical adjustment, despite the difference in their initial strength; glazing of ALD can improve material strength and eliminate the influence of the incorporated defect, but this is not the case for LD. Therefore, our recommended moment for ALD defect incorporation is after crystallization and before the glaze firing (*cig*); while for LD, defect incorporations before crystallization (*ic*, *icg*) are also acceptable; though glazing after defect incorporation can lead to an intra-oral start of the restoration at the highest strength, such initial strength of the restoration will decrease if a necessary adjustment has to be performed after the glaze firing. Further studies are needed to reveal the effect of surface treatments after necessary clinical adjustment, such as polishing and reglazing, on the flexural strength of ALD restorations. Another limitation of this study is that the defects incorporated do not completely simulate the real defects introduced on ceramic restorations in the clinical situation, because the shape and size of the defects and location of the adjustments for each case can be distinct from the others. When the size of the introduced flaw is too small or located in a low stress region, it may not have a noticeable effect on strength. However, in this study, only one defect was already sufficient to promote significant differences in the results. Future studies should be performed to investigate damages caused by the milling burs, as well as two- and three-body wear to investigate the influence of defect incorporation in simulated function with other mechanical properties for reinforced glass–ceramic restorations.

## Conclusions

According to the limitations of this study, it was possible to draw the following conclusions:

1. Advanced lithium disilicate showed lower flexural strength compared to a well-known lithium disilicate.
2. Considering the finishing technique, different from lithium disilicate, the advanced lithium disilicate must be crystallized and receive a glaze layer to achieve its highest flexural strength
3. Defects should not be incorporated after glaze firing. The less critical moment when a defect is incorporated occurs on crystalized restorations that will receive a glazer layer after
4. Defects incorporated in pre-crystallized advanced lithium disilicate and lithium disilicate can be healed during crystallization firing.

## Data Availability

The data will be available when requested to the corresponding author.
